# Real life management of patients hospitalized with multiple myeloma in France

**DOI:** 10.1371/journal.pone.0196596

**Published:** 2018-05-01

**Authors:** Charles Dumontet, Sandrine Couray-Targe, Marion Teisseire, Lionel Karlin, Delphine Maucort-Boulch

**Affiliations:** 1 Hematology Department, Hospices Civils de Lyon, Pierre-Bénite, France; 2 Université Lyon I, Villeurbanne, France; 3 DIM, Hospices Civils de Lyon, Lyon, France; 4 Hospices Civils de Lyon, Service de Biostatistiques, Lyon, France; 5 CNRS UMR 5558, Equipe Biostatistique Santé, Pierre-Bénite, France; Charles P. Darby Children's Research Institute, UNITED STATES

## Abstract

**Background:**

Patients with multiple myeloma included in prospective clinical trials are highly selected and therefore are expected not to be representative of the entire patient population. Additionally recommendations based on literature data and randomized trials are not systematically implemented in all patients. We sought to determine how patients hospitalized with a diagnosis of multiple myeloma are currently treated in France.

**Methods and findings:**

We performed a nation-wide search using the Programme de Médicalisation des Systèmes d’Information (PMSI) database which includes anonymous data for all patients hospitalized in France. We identified newly diagnosed cases in 2012 and analyzed the number and duration of hospital stays, coexisting conditions and treatment modalities with data available until the end of 2015. A diagnosis of multiple myeloma was determined for the first time during a hospitalization in France in 2012 in 6,282 patients (3,234 males and 3,048 females). The median age at diagnosis was 74 years (72 in males and 76 in females). A majority (55.3%) of patients were diagnosed and treated in a single heath center, including 37% in a university hospital and 52% in a non-university public hospital. Comorbidities potentially impacting on myeloma treatment were present in 57.5% of patients at diagnosis, and 15% had an associated diagnosis of another neoplasia. Intensive therapies with stem cell transplants were performed in 1033 patients (16% of total), the majority of which were aged less than 65 (881 patients, 85.3%). Stem cell transplants were performed more frequently in males while the distance between the site of residence and the transplant center had no impact on likelihood of receiving a transplant. Only 60% of patients less than 65 years old who were treated for their disease underwent intensification with stem cell transplant within the 4-year follow-up period.

**Conclusions:**

A large majority of patients hospitalized with a diagnosis of multiple myeloma are elderly, in particular females, and not eligible for transplants. Among the patients aged less than 65 and receiving therapy for their disease, 40% do not undergo transplants. These data emphasize the need for alternative therapies.

## Introduction

Multiple myeloma is classically considered to be the second most frequent hematological malignancy after lymphoma and ranks 14^th^ among all neoplasia in the SEER database, representing approximately 2% of all new cancer cases (https://seer.cancer.gov/data/). Incidence rates are reported to vary from 0.3 to 8.8 /100,000 in males (average 5.5) and 0.6 to 5.5/100,000 in females (average 3.7) within the European Union in 2012 (http://eco.iarc.fr/eucan/Cancer.aspx?Cancer=39). In 2012 the SEER incidence rate of myeloma in the USA was estimated to be 8.56/100,000 in males and 5.18/100,000 in females. In Great Britain and Sweden the age-adjusted incidence was estimated to be in the order of 5/100,000 [1]. Since the median age at diagnosis is reported to be in the order of 69 years it has been suggested that the recent increase of the incidence rate is mainly due to the modification of the age distribution in the entire population [2].

An increasingly important issue in oncology is the management of elderly patients with cancer. Many hematological malignancies such as myeloma are more frequently diagnosed after the age of 65 years, a commonly accepted age limit for intensive therapies such as stem cell transplants, although “fit” patients commonly benefit from this therapy until the age of 70. As elderly patients are often afflicted with significant comorbidities and more likely to develop iatrogenic complications, it is important to consider how elderly patients are treated in current practice. A better understanding of the proportion of elderly (and very elderly i.e. age > 80 years) patients afflicted with myeloma and the existence of associated medical conditions is thus essential for the choice of optimal therapy.

Access to the most efficient therapies is a key issue in patients with myeloma. High-dose therapy with stem cell transplant remains the gold standard for first line therapy and should theoretically be proposed to all patients under the age of 65 years who are sufficiently fit [3]. Novel agents have recently been approved for therapy of myeloma, in particular proteasome inhibitors, IMIDs and more recently monoclonal antibodies [4]. However few data are available regarding the actual use of these therapies in the real life setting. Quach et al. reported that bortezomib was effective and safe in the scope of an extended access programme yet with a significantly lower response rate than those expected in clinical trials [5]. Conversely the VESUVE study suggested that the real-life use of bortezomib provided survival outcomes similar to those observed in clinical trials [6]. Overall there is a continuous improvement in the outcome of patients with multiple myeloma although the most significant improvements have concerned patients under the age of 65 years [7].

Several epidemiological studies have analyzed the incidence and prevalence of neoplastic diseases in different age groups but few have associated the frequency and nature of the ensuing hospitalizations, geographical distribution of patients and types of treatment applied or health centers where patients were treated. In order to better describe the patient characteristics, hospitalization flow, treatment modalities and associated medical conditions of all patients diagnosed with multiple myeloma, we analyzed the PMSI database which provides exhaustive anonymous data for all patients hospitalized in public and private health centers in France.

## Methods

### The PMSI database

The PMSI (Programme de Médicalisation des Systèmes d'Information) databank comprises anonymous data for all hospital stays in France, including public and private health centers. It is a nation-wide diagnosis-related group based information system in which each hospital stay is coded by the attending physician and includes at least one main diagnosis and the relevant associated diagnoses [8]. Additional information includes the type of hospital, where the patient came from and went to after his stay. Some procedures such as radiotherapy and bone marrow transplantation are documented, as well as costly medications, such as the administration of bortezomib. Data were available starting in 2007 and until December 31, 2015 as of the writing of this manuscript. Hospital stays described in the PMSI database include conventional hospitalizations with overnight stays in the hospital as well as outpatient (or “ambulatory”) visits. This database does not however include data on patients seen only in consultation. Data regarding oral medications, stage of disease and response to therapy are not available.

Patients included in this study were identified using the diagnostic code C900 for multiple myeloma, either as the main or as an associated diagnosis. We chose patients for whom the code was used in 2012 for the first time, whether or not the patient had previously been hospitalized since 2007.

As there is no secondary validation of the diagnoses in the database there may be errors in the codification. To reduce excess diagnoses we sought cases for which there was an alternate diagnosis of hematological malignancy and an unexpected occurrence of the code C900 during one of the hospital stays. This allowed us to eliminate 382 patients for whom the diagnosis of multiple myeloma was unlikely. Conversely we did not have the means to determine those patients with myeloma and for whom this information was not present in the database. A recent publication by Palmaro et al. suggests that the diagnostic codes present in the PMSI database can be used with a good specificity to identify incident cases of myeloma [9].

### Geographical distribution

Geographical distribution of cases and patient flow was analyzed by administrative areas (the 96 “Départements” in metropolitan France). For each administrative area population characteristics were available through the national institute INSEE (https://www.insee.fr/fr/). We analysed the number of new cases diagnosed in each administrative area, the ratio of new cases to the total population of each area in 2012 and the proportion of patients under the age of 65 having required therapy who proceeded to stem cell transplant.

### Myeloma-related complications and comorbidities

Myeloma may be associated with a number of complications due to the disease itself or to therapy. Some patients present with these complications at diagnosis while other will present complications during the course of the disease. We defined the following categories: thrombo-embolic events, peripheral neuropathy, diabetes, infectious complications, myeloid insufficiency and renal failure. We also determined which patients had an associated diagnosis of second neoplasia.

### Patient hospitalization flow

To determine in which type of health center and in how many different centers patients were hospitalized during the course of their disease we determined the site and type of first hospitalization as well as subsequent stays during the follow-up for newly diagnosed patients in 2012. For those patients receiving bone marrow transplants we analyzed the distribution of patients in centers performing these procedures.

### Access to intensive therapy

In order to determine whether some patient characteristics increased or decreased likelihood to have access to high dose therapy with stem cell transplants we analyzed whether age, sex, place of residence (i.e. living in an administrative area with a center performing stem cell transplants) had an influence on the percentage of intensive therapies received by patients.

### Statistics

Continuous variables were summarized with median, quartiles, minimum and maximum, mean and standard deviation; quantitative variables were described with number and percentages. Percentages comparisons were performed with chi2 test. Analyses were performed using R-software (software 3.0.2, 2013) (ref: R Development Core Team. R: A Language Environment for Statistical Computing. Vienna, Austria. ISBN 3-900051-07-0. URL: http://www.R-project.org 2013.)

## Results

### Analyzed population

**Fig 1:**
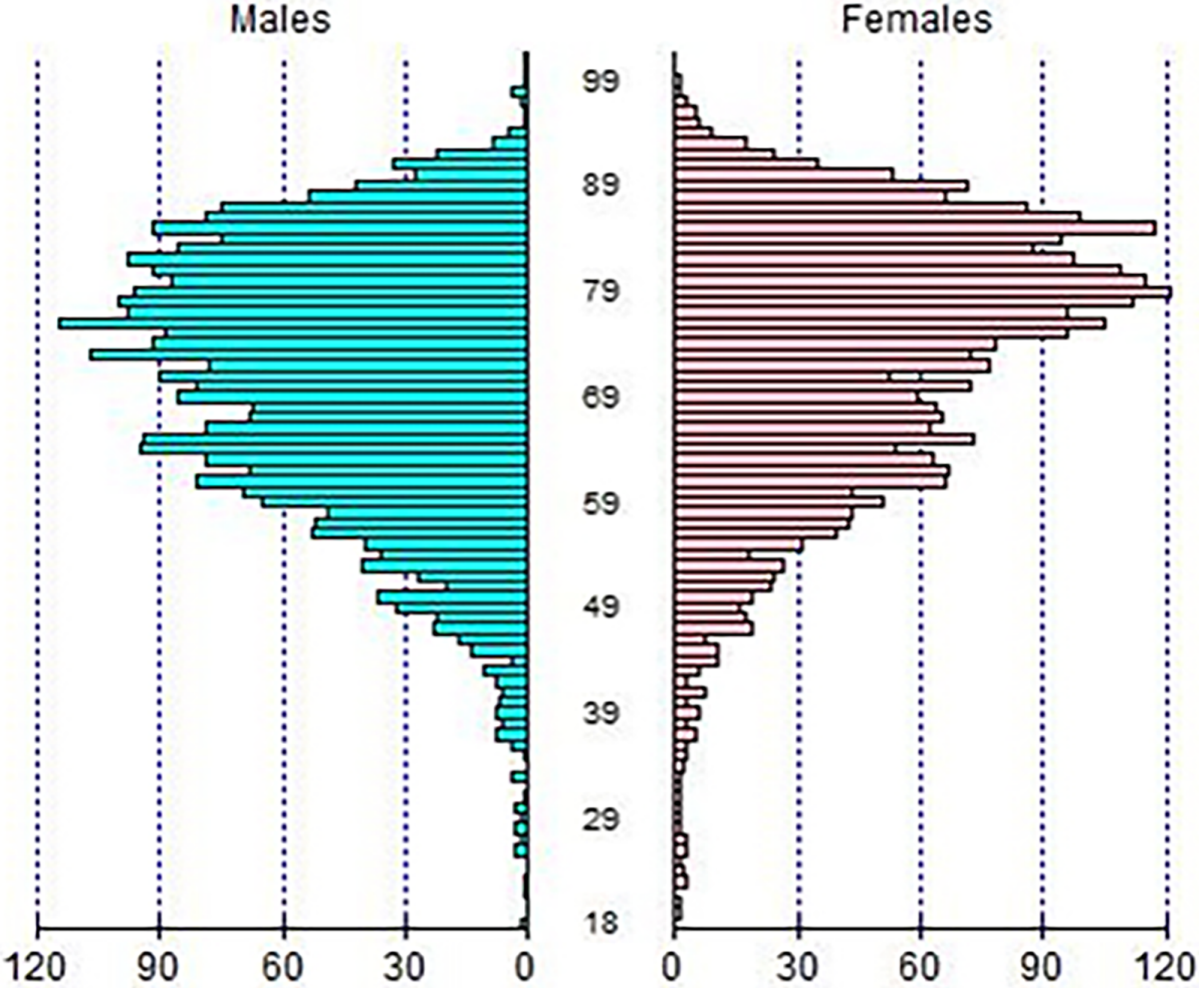
Age distribution by sex of patients newly diagnosed with multiple myeloma during a hospital stay in France in 2012.

In 2012, a total of 6,282 patients for whom a diagnosis of multiple myeloma was determined for the first time were hospitalized in France, representing an incidence rate of 1/10,000 (population of 65.68 millions in 2012). The number of prevalent cases (defined by all patients with a C900 code, including those patients who had previously been hospitalized with this diagnosis in previous years) was in the order of 16,000. The main patient characteristics are indicated in Table 1. There were 3,234 males and 3,048 females (sex ratio of 1.06). The median age at diagnosis was 74 years (72 in males, range 18–107, and 76 in females, range 19–99) (Table 1 and Fig 1). A majority of patients (72.1%) were aged 65 years or more (68.9% of all male patients and 75.4% of all female patients) and 28.4% were aged over 80 (24.8% of males and 32.2% of females) (Fig 1).

**Table 1:** Characteristics of incident cases of myeloma hospitalized for the first time in 2012 in France.

	Males Values (%)	Females Values (%)	Total Values (%)
	3234 (51.5)	3048 (48.5)	6282 (100)
Age			
Mean (SD)	70.6 (12.6)	73.0 (12.6)	71.8 (12.6)
Median (min-max)	72 (18–107)	76 (19–99)	74 (18–107)
< 65 yrs	1006 (31.1)	749 (24.6)	1755 (27.9)
65–80 yrs	1427 (44.1)	1319 (43.3)	2746 (43.7)
>80 yrs	801 (24.8)	980 (32.2)	1781 (28.4)
Geographical area at first hospitalization			
Same as residence*	2478 (76.6)	2369 (77.7)	4847 (77.2)
Different from residence	756 (23.4)	679 (22.3)	1435 (22.8)
Health center at first hospitalization			
University hospital	1077 (33.3)	945 (31.0)	2022 (32.2)
Non-university public hospital	1453 (44.9)	1430 (46.9)	2883 (45.9)
Cancer treatment center	64 (2.0)	73 (2.4)	137 (2.2)
Other private	521 (16.1)	461 (15.1)	982 (15.6)
Other	119 (3.7)	139 (4.6)	258 (4.1)

* “same as residence” is meant as in the same administrative area

### Associated medical conditions at first visit

We found at least one medical condition potentially related to myeloma or impacting on myeloma treatment in 57.5 of patients at diagnosis (Table 2). These included infectious (14.5%), renal (15.2%), cytopenic (14.5%) and metabolic (14.5%) conditions or complications of disease. Thrombo-embolic (2.7%), skeletal (0.7%) and peripheral neuropathic (0.7%) were reported less frequently. Comorbidities were as expected more frequent in elderly patients with a median of 2 associated conditions in patients under the age of 65, 3 in patients between 65 and 80 and 4 for those aged 80 or more. There were no differences in the rate of comorbidities between men and women. A total of 1,092 diagnoses of other neoplasia were declared in the database for 931 patients (15% of all patients)(Table 3).

**Table 2:** Medical conditions reported at diagnosis in patients with a hospital diagnosis of myeloma in 2012.

	Presence	males	females	total
		N = 3234	N = 3048	N = 6282
Renal	0	2693 (83.3%)	2635 (86.5%)	5328 (84.8%)
	1	541 (16.7%)	413 (13.5%)	954 (15.2%)
Infection	0	2780 (86.0%)	2594 (85.1%)	5374 (85.5%)
	1	454 (14.0%)	454 (14.9%)	908 (14.5%)
Metabolic	0	2730 (84.4%)	2641 (86.6%)	5371 (85.5%)
	1	504 (15.6%)	407 (13.4%)	911 (14.5%)
Myeloid toxicity	0	2805 (86.7%)	2565 (84.2%)	5370 (85.5%)
	1	429 (13.3%)	483 (15.8%)	912 (14.5%)
Vascular	0	3147 (97.3%)	2967 (97.3%)	6114 (97.3%)
	1	87 (2.7%)	81 (2.7%)	168 (2.7%)
Peripheral neuropathy	0	3206 (99.1%)	3031 (99.4%)	6237 (99.3%)
	1	28 (0.9%)	17 (0.6%)	45 (0.7%)
Bone lesions	0	3213 (99.4%)	3023 (99.2%)	6236 (99.3%)
	1	21 (0.6%)	25 (0.8%)	46 (0.7%)

**Table 3:** Associated diagnoses of neoplasia in patients diagnosed with myeloma in 2012.

Localization	number of patients	%
metastases	371	5.9
digestive tract	170	2.7
urological	131	2.1
skin	124	2
thoracic	78	1.2
bone	67	1.1
breast	55	0.9
upper airway	27	0.4
CNS	19	0.3
soft tissue	17	0.3
gynecological	16	0.3
endocrine	12	0.2
eye	5	0.1

### Patient hospitalization flow

Patients diagnosed with myeloma were hospitalized a median number of 15 times during their follow-up. The median time between the first and the last hospital stay for all patients newly diagnosed in 2012 was 15.8 months. During the follow-up period of 4 years a total of 1,526 (24.3%) patients were reported to have died during a stay at the hospital, at a median age of 77 years for males and 79 years for females. There were no data available for patients who had died outside of the hospital. The duration of the hospitalization stays was associated with age with a mean duration of 3.01 days in patients under the age of 65, 4.34 days for those aged between 65 and 80 and 7.66 days for those aged 80 or more.

A majority (55.3%) of patients were diagnosed and treated in a single heath center, among which 37% were treated in a university hospital, 52.3% in a non-university public hospital and 10.7% in other institutions (anticancer centers, private clinics/hospitals, other). Patients under 65 years of age were more frequently treated in university hospitals (62.8%) than older patients (34.8% between the ages of 65 and 80 and 24.7% above 80 years of age).

### Treatment

With a median follow-up of 506 days after their first hospitalization with a diagnosis of multiple myeloma, 4,218 (67.1%) of patients diagnosed in 2012 had received therapy during hospitalization. In patients aged less than 65 years, 82.1% received therapy at the hospital while73.9 of patients aged between 65 and 80 received therapy and only 41.9% older than 80. This is an underestimation of the total number of patients effectively treated since it does not take into account exclusively oral therapy which is commonly used in elderly patients with myeloma. A majority (66%) of all patients receiving treatment at the hospital did so in the outpatient setting with a median time between diagnosis and the first treatment of 35 days. 54.7% of all hospital stays included the administration of anticancer agents.

Bortezomib was administered in 4,221 patients, or 67.2% of all patients. Bortezomib was widely used until the age of 80 since it was administered to 70% of patients under the age of 65 having required therapy and 71% of patients between the ages of 65 and 80 having required therapy, but only 51% of the more elderly patients who were treated in the hospital.

Overall 496 patients underwent dialysis during the follow-up period, including 31.2% of which were performed during the first hospital stay. Approximately 40% of the dialysed patients were women, and the majority was aged between 65 and 80 years (144 were under 65 year-old, 262 between the ages of 65 and 80, and 90 were over 80 year-old). 562 patients (8.9%) received radiotherapy during the follow-up period. These included 324 males (57.7%) and 238 females (42.3%) with a median age of 67 years for males and 70 years for females.

A total of 1,157 intensive therapies with stem cell transplants were performed in 1,033 patients (or 16% of the entire patient population), including 927 patients who received a single transplant and 106 who received two or more. The median duration of hospitalization for this procedure was 18 days. The median delay between the first hospital stay with a diagnosis of myeloma and stem cell transplant was 161 days. The majority of patients (881 patients, 85.3%) receiving a stem cell transplant were aged less than 65 years, with a significant difference between males (614 patients, 59.4% of all patients receiving stem cell transplant) and females (419 patients, 40.6% of all patients, p<0,001). This was at least partly attributable to the fact that female patients were generally older than male patients. However among patients under the age of 65 and having required therapy the proportion of patients who proceeded to a stem cell transplant was higher among males (63.2%) than in females (58.3%, p<0.05).

**Fig 2:**
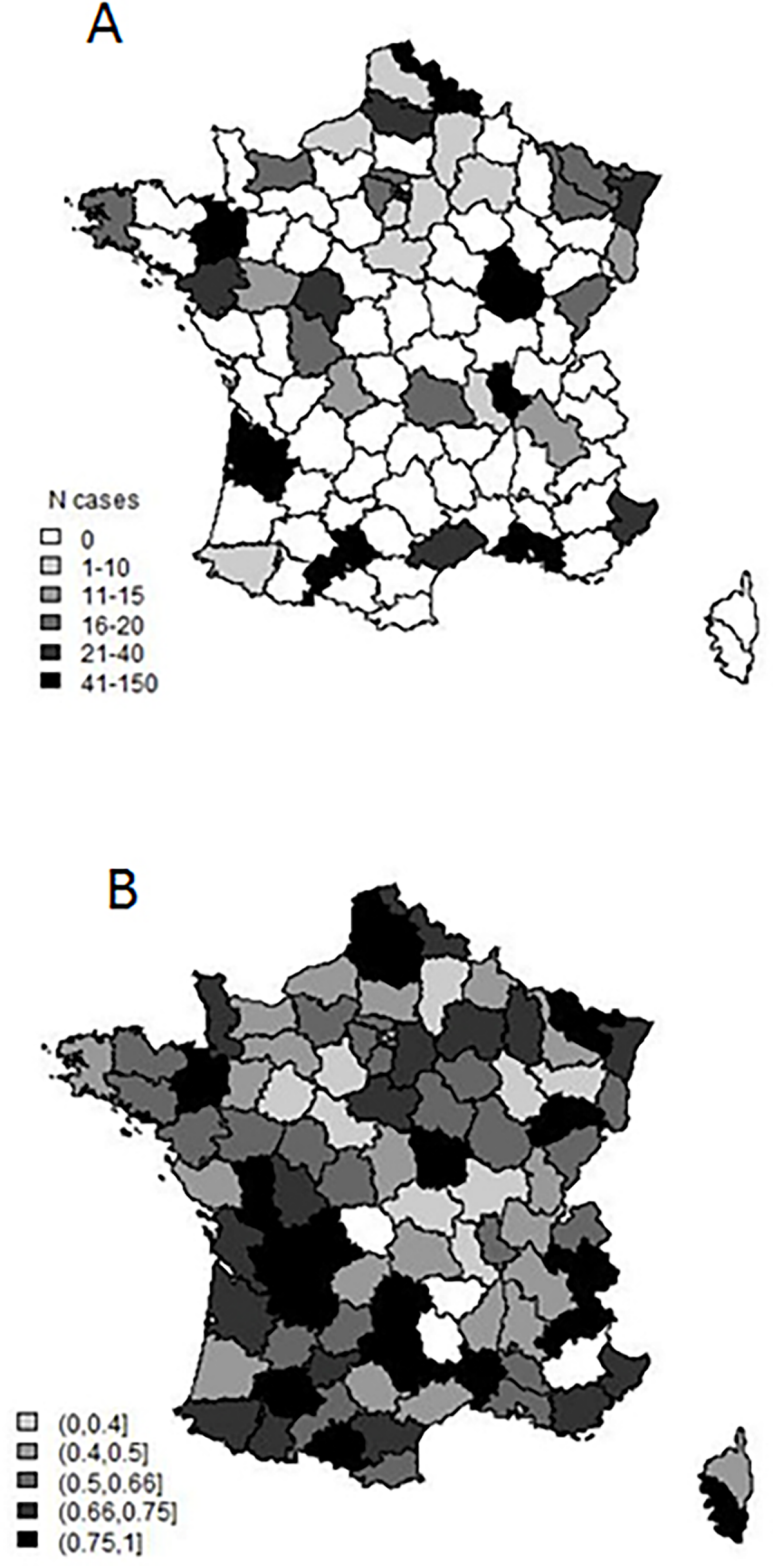
Geographical distribution of stem cell transplants in France in patients newly diagnosed with myeloma in 2012. Fig 2A. Geographical distribution of stem cell transplants performed in patients less than 65 years old at diagnosis during their follow-up. Stem cell transplants were performed in 39 administrative areas or “Départements”. Fig 2B. Probability of proceeding to a stem cell transplant according to place of residence in patients less than 65 years old and having received therapy in the hospital for their disease.

Stem cell transplant procedures were performed in 39 administrative areas (Fig 2A). The likelihood to proceed to a stem cell transplant for a patient aged less than 65 years diagnosed with myeloma in 2012 and having required therapy for his/her disease was similar whether or not the patient lived in an are in which stem cell transplants were performed (59.2% of patients living in an area with a transplant center and 55.9% of patients living in an area with no transplant center (p = 0.24)(Fig 2B).

## Conclusion

Our study identified 6,282 new cases of patients hospitalized with a diagnosis of myeloma in 2012, or an incidence of approximately 1/10,000. Limitations to this study include the fact that some cases may have not properly been identified during hospitalization. Another cause of error is the fact that patients treated before 2007 and who had not been hospitalized until 2012 were considered as “new” diagnoses. Excess diagnosis in the case of multiple myeloma may be due to the fact that patients with MGUS have been classified with authentic myeloma while patients who were not hospitalized with a diagnosis of myeloma were not taken into account. Elderly patients with other severe diseases may not have been hospitalized or the diagnosis of myeloma may have been ignored. Notwithstanding these caveats the incidence values observed in our study are similar to those reported for France in 2012 in the Euracan database (6.6/100,000) and similar to those observed in other countries such as Norway (http://eco.iarc.fr/eucan/Cancer.aspx?Cancer=39). While myeloma has been reported to be more frequent in blacks than whites we did not have access to ethnic characteristics of patients [10].

An important observation in terms of treatment modalities is the age distribution at diagnosis in newly diagnosed patients. The majority of patients could be considered as elderly (43.7% between the ages of 65 and 80) or very elderly (28.4% age greater than 80) with a higher median age at diagnosis in females. This may represent an underestimation of the actual median age at diagnosis since younger patients are more likely to have been hospitalized with the correct diagnosis then the very elderly. In 2016 18.8% of the total population in France was aged 65 or more. According to Eurostat projections, in 2060 individuals older than 65 and 80 could represent 30% and 12% of the entire European population, respectively. This observation has several short and medium term implications. Elderly patients are not in their vast majority eligible for intensification therapy, but clearly benefit from agents such as IMIDs or bortezomib [11,12] and are likely to benefit from recently approved monoclonal antibody therapies. Blommestein et al. analyzed several cohorts of elderly patients with myeloma and estimated that the cost for a quality adjusted life year could be in the order of 35,000 euros [13]. It should thus be expected that the number of newly diagnosed cases of myeloma will increase significantly and that the cost associated with the treatment of this disease will increase accordingly in the years to come.

Comorbidities represent a major issue in the treatment of patients with multiple myeloma. These may limit the choice of therapies depending on the expected toxicities, enhance the risk of complications and have a major influence on the decision to treat the disease with curative intent or to adopt a palliative approach. Offidani et al. have proposed a Vulnerability Score in order to help tailor patient therapy [14]. In our study a majority of patients (57.5%) presented with associated medical conditions at diagnosis of myeloma and the frequency of these conditions increased during follow-up. In particular infectious episodes were identified in 14.5% of patients at diagnosis and up to 50.9% during the following hospital stays. Renal insufficiency was present in 14.5% of patients at diagnosis and a total of 32.9% patients presented this complication at some time during follow-up and 8% required dialysis. Fifteen percent of patients with an initial diagnosis of myeloma also had an associated diagnosis of another neoplastic disease, the treatment of which is likely to have an impact on the treatment of myeloma.

Our study shows that among the newly diagnosed patients aged under 65 years who received therapy, 60.4% undergo transplant during the follow-up period of this study. This suggests that a significant fraction of patients for whom transplant is an option to be considered do not undergo high dose therapy. This may be due to the fact that some patients refuse high dose therapy, that such therapy is not offered or that there is some co-existing condition which precludes high dose therapy. Overall only 16% of all patients newly diagnosed with myeloma in 2012 proceeded to high dose therapy during follow-up, emphasizing the need for alternative efficient therapies.

Real life studies are increasingly being used to determine both the actual status of unselected patients outside of the scope of clinical trials, and compare treatment modalities in respect to recommendations and guidelines. In a recent study Palmaro et al. found that among myeloma patients receiving IMIDs and were thus at high-risk of thrombo-venous embolic events up to half of patients did not receive proper prophylactic therapy [15]. In a single institution analysis of 621 myeloma patients Rios-Tamayo et al. found that comorbidities (which are classical exclusion criteria for clinical trials) played a critical role in early mortality [16]. Mian et al. analysed 949 patients followed over a 35 –year period and concluded that the availability of novel agents such as bortezomib and IMIDs had improved prognosis both in transplant-eligible and transplant-ineligible patients [17]. Leleu et al. performed a large multicentric study to analyze risk factors of thrombo-embolism in myeloma patients receiving IMIDs, showing discrepancies in prophylactic approaches [18]. Using a SEER-based registry, Winn et al. found that high dose therapy with stem cell transplant improved outcome of patients older than 65 in the real-life setting [19].

In conclusion this study reports for the first time real life data regarding patients hospitalized for myeloma at a nation-wide scale. Important findings are the fact that the majority of patients are aged 65 or more, that female patients are older and less likely to proceed to transplant and that only a small minority of all patients will proceed to stem cell transplant. There will thus be an increasing need for novel effective and less toxic therapies in the years to come.
